# First Selective Renal Angiogram in a Nigerian With Flash Pulmonary Edema and Suspected Renal Artery Stenosis

**DOI:** 10.7759/cureus.85177

**Published:** 2025-06-01

**Authors:** Olurotimi J Badero, Pelumi V Ajayi, Bamikole Osibowale

**Affiliations:** 1 Interventional Cardiology, Iwosan Lagoon Hospitals, Lagos, NGA; 2 Interventional Cardiology, Division of Cardio-Nephrology, Cardiac Renal & Vascular Associates, Jackson, USA; 3 General Medicine, Iwosan Lagoon Hospitals, Lagos, NGA

**Keywords:** coronary artery disease, flash pulmonary edema, nigeria, renal angiogram, renal artery stenosis

## Abstract

To the best of our knowledge, we report the first selective renal angiogram performed in Nigeria. The patient presented with sudden-onset shortness of breath, elevated blood pressure, flash pulmonary edema, and left ventricular systolic dysfunction and was eventually found to have multi-vessel coronary artery disease, managed surgically with coronary artery bypass grafting. This report illustrates the technique of performing selective renal angiogram, particularly in cases of suspected renal artery stenosis, and underscores the significance of a multidisciplinary approach in optimizing patient outcomes.

## Introduction

Selective renal angiogram remains the definitive and gold standard diagnostic modality for renal artery diseases despite the technological advancement in non-invasive diagnostic methods [1]. It is, however, not routinely done because of invasiveness, cost, and possible complications. Indications for renal vascular evaluation include clinical suspicion of renal artery stenosis, such as accelerated, malignant, or resistant hypertension, unexplained atrophic kidney or discrepancy in kidney sizes, hypertension in the young, and flash pulmonary edema, among others [2].

Flash pulmonary edema is a clinical syndrome characterized by the abrupt onset of acute left ventricular systolic dysfunction, typically with preserved cardiac output, resulting in rapid and severe fluid accumulation within the pulmonary interstitial and alveolar spaces. This condition, also referred to as Pickering syndrome (first described by Dr. Thomas Pickering in 1988), arises from a complex interplay between cardio-renal dysfunction and impaired fluid regulation. Precipitating factors include acute myocardial ischemia, left ventricular diastolic dysfunction, acute elevations in blood pressure or intravascular volume, and bilateral renal artery stenosis [3].

Renal artery stenosis is the predominant etiology of secondary hypertension, frequently manifesting as treatment-resistant hypertension. Notably, the administration of angiotensin-converting enzyme inhibitors (ACEIs) or angiotensin receptor blockers (ARBs) may exacerbate hypertension in these patients, leading to elevated risks of cardiovascular and renal morbidity and mortality. Atherosclerotic disease accounts for nearly 90% of renal artery stenosis cases, with a heightened prevalence observed in individuals with diabetes mellitus, dyslipidemia, and coronary artery disease [4]. Based on the initial evaluation of our patient who presented with malignant hypertension, acute flash pulmonary edema, and cardiomyopathy, following prompt diuresis, the decision was made to schedule him for a planned cardiac catheterization and bilateral selective renal angiogram to rule out ischemic etiology of his cardiomyopathy and renal artery stenosis. Drive-by renal angiogram that sometimes occurs during cardiac catheterization is also considered a reasonable approach, given the increasing prevalence of renal artery stenosis and its reported coexistence with coronary artery disease [5,6].

The availability and utilization of advanced diagnostic and interventional cardiology procedures are continuously evolving in developing countries like ours. To the best of our knowledge, this case report describes the first documented selective renal angiogram performed in Nigeria, highlighting the technical aspects of the procedure and its role in the diagnostic workup of a patient with suspected renovascular hypertension presenting with flash pulmonary edema, who was ultimately found to have significant coronary artery disease.

## Case presentation

A 65-year-old man with a history of hypertension, dyslipidemia, and gastroesophageal reflux disease (GERD) with poor medication compliance was admitted to our facility with shortness of breath, which started three days prior to presentation. He was resting at home when he developed sudden-onset shortness of breath and a feeling of drowning. There was an associated productive cough of whitish sputum with no fever, chest pain, or leg swelling. He was taken to the nearest hospital by his family members, where his blood pressure was notably elevated with malignant hypertension and features of pulmonary edema on chest X-ray (CXR). He was subsequently treated with intravenous diuretics (furosemide) with clinical improvement. He was transferred to our facility for further management.

Physical examination and vitals were within normal limits. Cardiac workup included an electrocardiogram (EKG) that showed normal sinus rhythm, left ventricular hypertrophy (LVH), and T-wave inversions in the anterolateral leads. Echocardiogram revealed moderate left ventricular global systolic dysfunction (left ventricular ejection fraction: 36%), moderate left atrial enlargement (LAE), mild to moderate mitral regurgitation, mild pericardial effusion, and reduced tricuspid annular plane systolic excursion (TAPSE) (14.9 mm). His lipid profile was abnormal with elevated total cholesterol and low-density lipoprotein (LDL) of 215 mg/dl (desirable level: <200 mg/dl) and 170.4 mg/dl (optimal level: <100 mg/dl), respectively, while his high-density lipoprotein (HDL) was 34 mg/dl (L) (optimal level: >60 mg/dl) and triglyceride was 53 mg/dl (desirable level: <150 mg/dl).

An assessment of systolic congestive heart failure with moderate cardiomyopathy and suspected renal artery stenosis was made. He was scheduled for combined right and left heart catheterization to rule out ischemic etiology and assess pulmonary pressures as well as renal angiogram to rule out renal artery stenosis.

Procedural technique

Left and Right Heart Catheterization

Left and right heart catheterization was performed via transfemoral access, under moderate conscious sedation of 1 mg midazolam and 25 mcg fentanyl administered by the nurse. Arterial and venous accesses were obtained with 6- and 7-French sheaths, respectively, using the sterile Seldinger technique after administering 1% lidocaine for local anesthesia. The sheaths were placed in the left common femoral artery and vein, respectively.

A 7-French Swan-Ganz catheter was then used to perform right heart catheterization, including oxygen saturation, pressure measurement, and cardiac output measurement by the Fick method. The Swan-Ganz catheter was removed, and Judkins left 4 (JL4) and Judkins right 4 (JR4) diagnostic coronary catheters were used to perform diagnostic coronary angiography in multiple orthogonal views. A 6-French pigtail catheter was advanced across the aortic valve into the left ventricular cavity, and pressures were recorded. Left ventriculogram was performed in the standard 30-degree right anterior oblique projection, and under continuous monitoring, the catheter was withdrawn from the left ventricular cavity into the aorta.

Bilateral Selective Renal Angiogram

**Figure 1:**
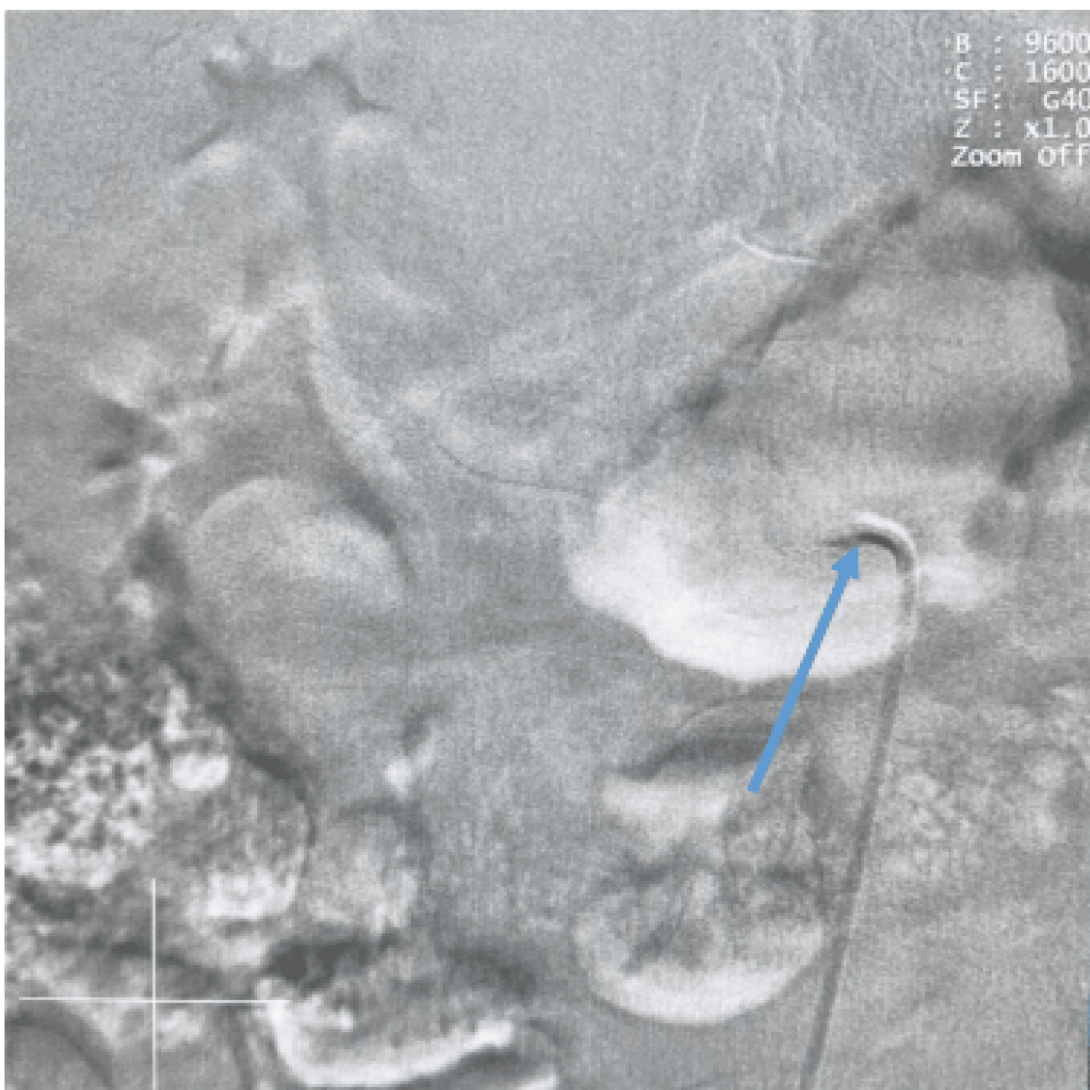
Cannulation of the right renal artery Cannulation of the right renal artery before dye injection with the tip of the catheter engaged at the ostium of the artery (blue arrow)

**Figure 2:**
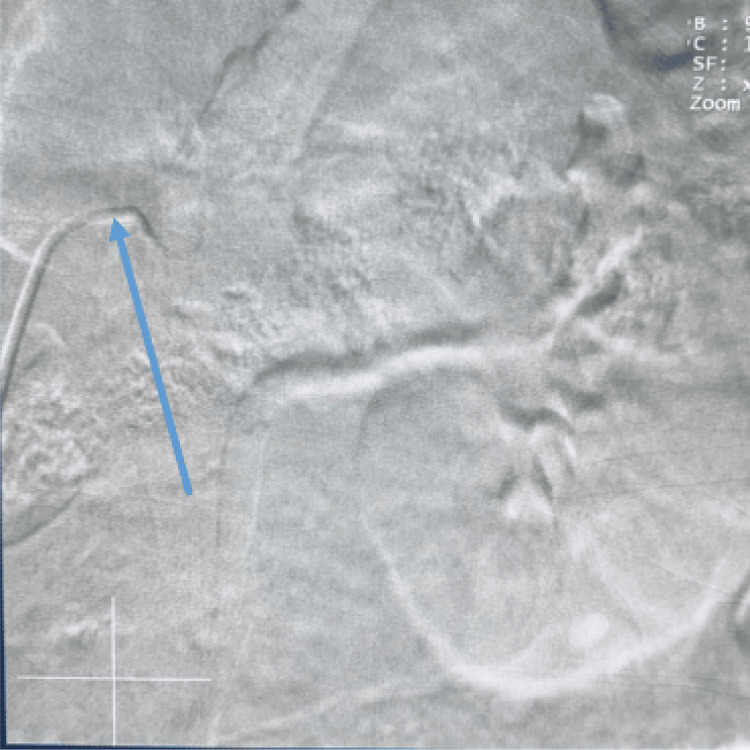
Cannulation of the left renal artery Cannulation of the left renal artery before dye injection with the tip of the catheter engaged at the ostium of the artery (blue arrow)

The pigtail catheter was exchanged for a 6-French JR4 catheter, which was advanced into the abdominal aorta and was used to selectively engage the right and left renal arteries at the level of the L1-L2 vertebral bodies. The left renal artery was selectively cannulated slightly higher than the right. Bilateral selective renal angiogram was then performed accordingly. The catheter was then removed over a 0.035 wire (Figures 1-2).

A completion femoral angiogram was performed to confirm adequacy for closure device deployment. At the end of the procedure, all wires, catheters, and sheaths were removed, and the arteriotomy and venotomy sites were sealed with manual pressure applied successfully. The procedure was completed without any immediate complications.

Findings

Bilateral Renal Angiogram Findings 

**Figure 3:**
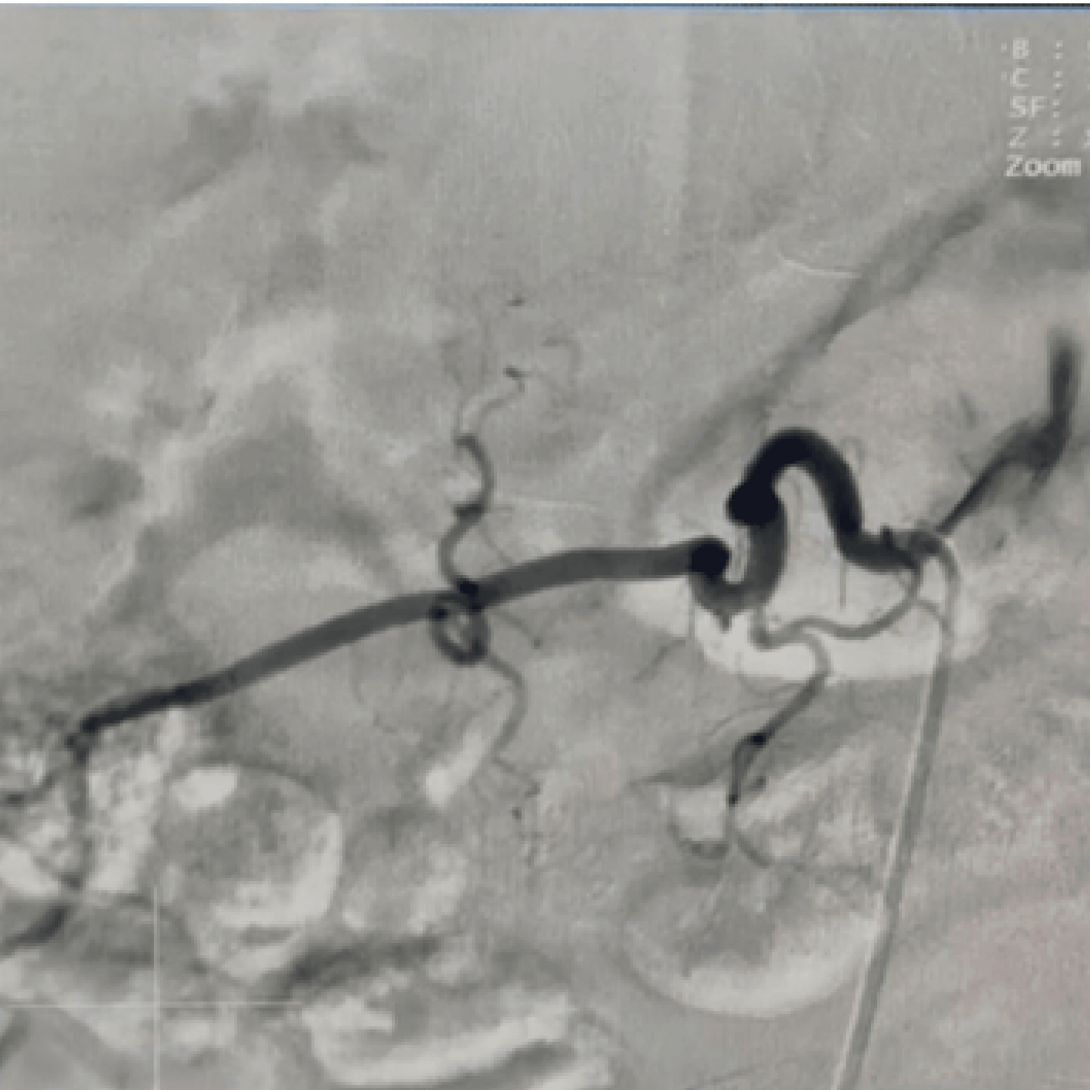
Right renal angiogram Cannulation of the right renal artery after dye injection highlighting the right renal artery with no evidence of stenosis

The right renal artery was normal in caliber, bifurcating just proximal to the renal hilum, supplying branches to the respective poles. The right renal artery had no evidence of stenosis or focal dilatation. There was no evidence of any accessory renal artery noted (Figure 3).

**Figure 4:**
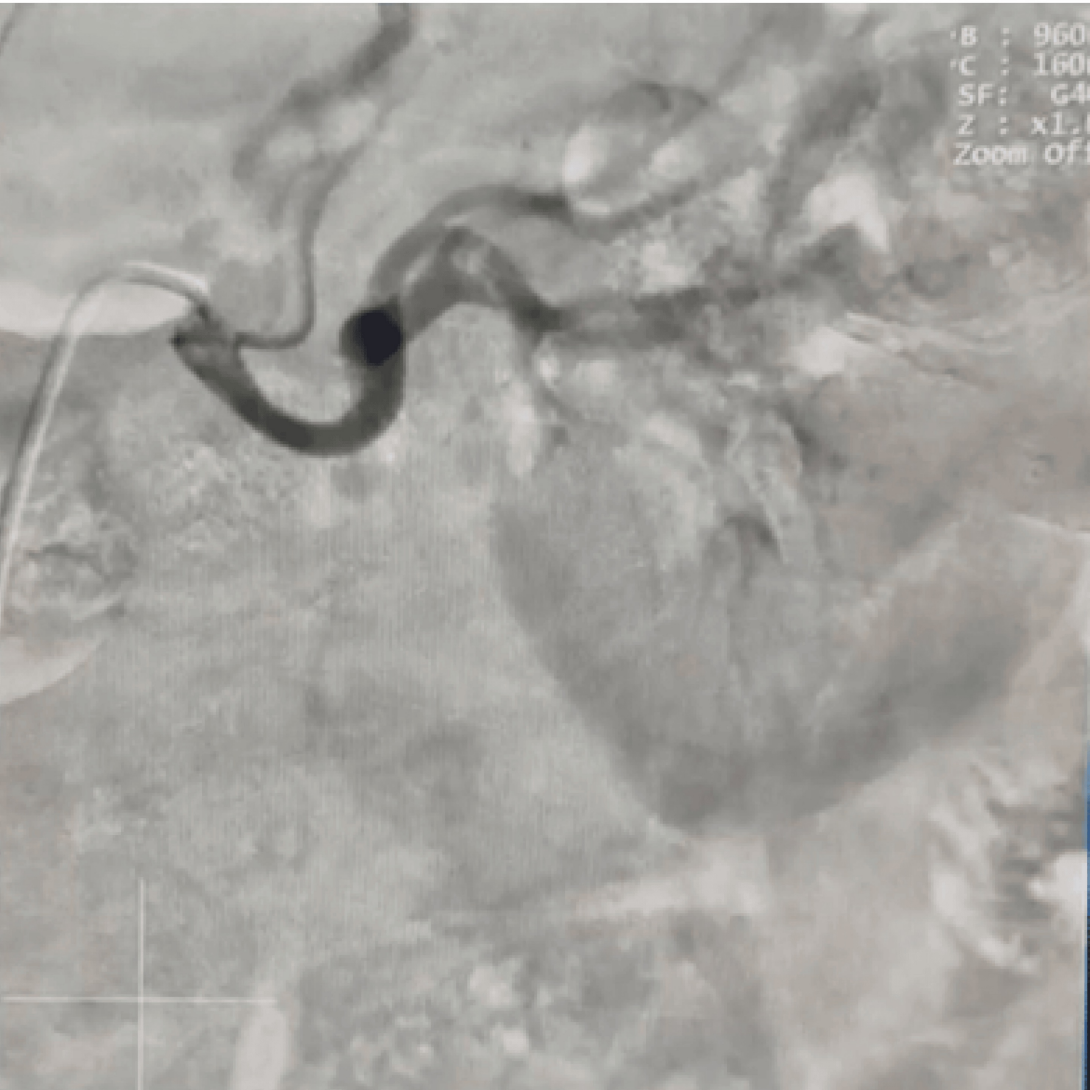
Left renal angiogram Cannulation of the left renal artery after dye injection highlighting the left renal artery with no evidence of stenosis

The left renal artery was normal in caliber, bifurcating just proximal to the renal hilum, supplying branches to the respective poles. The left renal artery had no evidence of stenosis or focal dilatation. There was no evidence of any accessory renal artery noted (Figure 4).

Left Ventriculogram

Left ventricular ejection fraction was estimated at 35%, consistent with recent transthoracic echocardiographic findings. Left ventricular pressure was measured at 130/32 mmHg, with an elevated end-diastolic pressure (EDP) of 34 mmHg. Aortic pressure was recorded at 134/45 mmHg, with a mean arterial pressure of 71 mmHg. No transvalvular gradient was observed across the aortic valve, indicating no significant aortic stenosis.

Coronary Angiographic Findings

**Figure 5:**
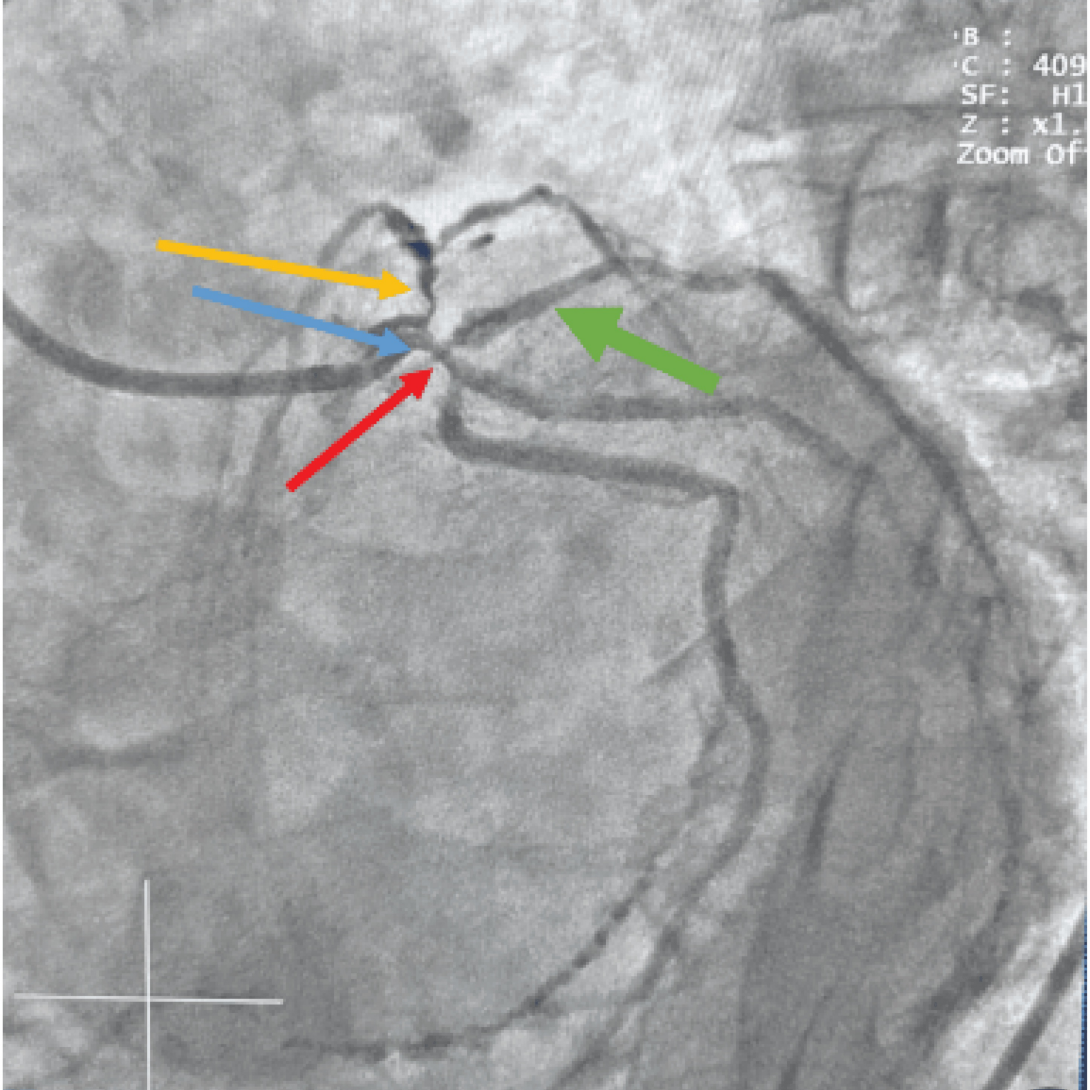
Complex significant trifurcation disease involving the distal left main coronary artery (blue arrow), ostial left anterior descending artery (yellow arrow), ostial left circumflex artery (red arrow), and ostial ramus intermedius artery (green arrow)

The left main coronary artery was short-segmented, with a normal-caliber vessel and a trifurcation into the left anterior descending, ramus intermedius, and left circumflex arteries. A 99% stenosis was identified at the distal trifurcation of the left main artery (Figure 5).

The left anterior descending artery was a normal-caliber, transapical vessel giving rise to moderate-caliber diagonal branches and septal perforators. A critical 99% stenosis was noted at the ostium of the left anterior descending artery. The ramus intermedius artery was a normal-caliber vessel with a 99% ostial stenosis. The left circumflex artery was a non-dominant, normal-caliber vessel giving rise to appropriately sized obtuse marginal branches. A 90% ostial stenosis was identified, with an additional moderate stenosis noted in the distal segment of the left circumflex artery.

**Figure 6:**
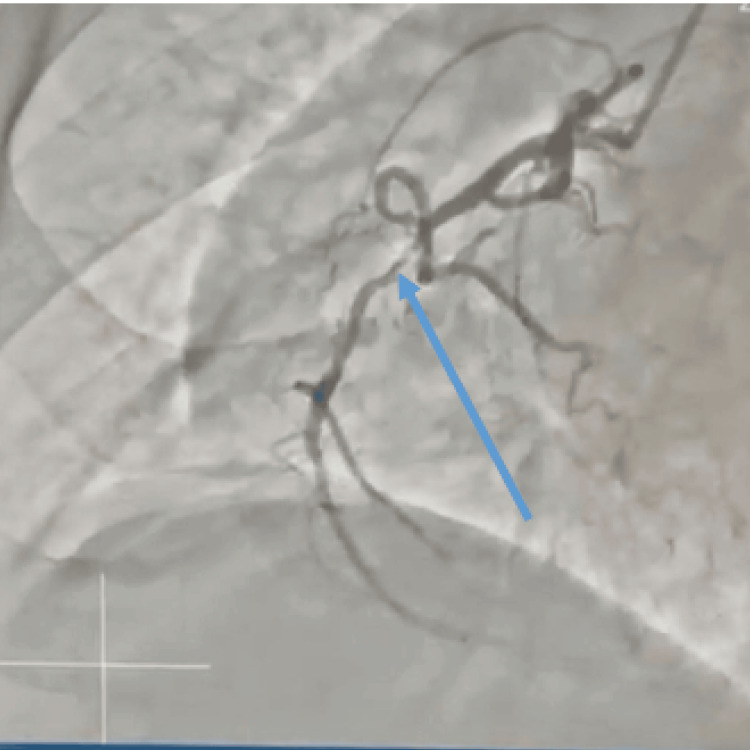
Right coronary artery with significant mid-segment stenosis (blue arrow)

The right coronary artery was a dominant, normal-caliber vessel giving rise to the right posterior descending artery and right posterolateral branches. The right coronary artery demonstrated an 80% stenosis in the mid-segment. The right posterior descending artery was found to be occluded, with collateral circulation from the left circumflex artery supplying the distal vessel (Figure 6).

Right Heart Catheterization Findings

Pressure measurement showed systolic and diastolic pulmonary capillary wedge pressure of 11/12 with a mean of 11, pulmonary artery systolic and diastolic pressure of 29/14 with a mean of 19, right ventricular systolic and diastolic pressure of 29/5 with a mean of 6, and right atrial systolic and diastolic pressure of 8/6 with a mean of 5 (Table 1).

**Table 1:** Hemodynamic pressure measurements

Parameter	Systolic/diastolic (mmHg)	Mean (mmHg)	Interpretation
Pulmonary capillary wedge pressure	11/12	11	Normal
Pulmonary artery pressure	29/14	19	Mildly elevated; borderline pulmonary hypertension
Right ventricular pressure	29/5	6	Normal systolic, mildly low diastolic
Right atrial pressure	8/6	5	Within normal limits

The femoral artery oxygen saturation was 90.7%, the pulmonary artery oxygen saturation was 73.6%, and the right atrial oxygen saturation was 74.3% (Table 2). The calculated cardiac output by the Fick method was 4.8 L/min, and the cardiac index was 2.7 L/min/m^2^. The systemic vascular resistance was 1440 dyn·s·cm^−5^, and the pulmonary vascular resistance was 133.3 dyn·s·cm^−5^ (Table 3).

**Table 2:** Oxygen saturation

Sampling site	Oxygen saturation (%)	Interpretation
Femoral artery	90.7	Slight low; may reflect mild hypoxemia
Right atrium	74.3	Within expected venous range
Pulmonary artery	73.6	No significant step-up; no left-to-right shunt

**Table 3:** Cardiac function parameter

Parameter	Value	Interpretation
Cardiac output (Fick method)	4.8 L/min	Within normal range (4-8 L/min)
Cardiac index	2.7 L/min/m^2^	Normal (2.5-4.0 L/min/m^2^)
Systemic vascular resistance	1440 dyn·s·cm^−5^	Elevated (900-1400 dyn·s·cm^−5^); may reflect systemic afterload
Pulmonary vascular resistance	133.3 dyn·s·cm^−5^	Elevated (20-130 dyn·s·cm^−5^); may reflect mild pulmonary hypertension

The pre-procedure creatinine was 0.9 mg/dl, with an estimated glomerular filtration rate (eGFR) of 104 ml/min/1.72 m^2^ per the Chronic Kidney Disease Epidemiology Collaboration (CKD/EPI), and the post-procedure creatinine after 48 hours was 1.0 mg/dl, with an eGFR of 91 ml/min/1.72 m^2^.

Following a heart team discussion, the patient was appropriately referred to coronary artery bypass grafting. He eventually had a left internal mammary graft to the left anterior descending artery and saphenous vein grafts to the ramus intermedius, left circumflex, and right coronary arteries. He did well postoperatively and was scheduled for close monitoring as an outpatient follow-up.

## Discussion

This case report documents the first selective renal angiogram performed in Nigeria in a patient presenting with flash pulmonary edema and suspected renal artery stenosis. 

The history of renal angiography dates back to 1929, when Dos Santos pioneered translumbar aortography, later refined by Peirce through arterial catheterization and further advanced by Seldinger's technique in 1953. Since then, renal angiography techniques have undergone significant improvements, enhancing diagnostic and therapeutic precision in renal vascular diseases [7].

Among the various diagnostic modalities available for evaluating renal vascular pathologies, renal angiography remains the gold standard, offering both diagnostic and therapeutic capabilities. It provides detailed visualization of the renal arteries and their branches and morphological features while also facilitating endovascular interventions during the procedure [1].

Angiographic images are generated either via conventional X-ray films following intravenous contrast administration or through digital subtraction angiography (DSA). DSA employs an X-ray source and an image intensifier, capturing sequential images. An initial pre-contrast image (mask) is subtracted from subsequent post-contrast images, allowing the selective visualization of the contrast-filled vasculature while eliminating background interference [1].

While the renal angiogram did not reveal any significant stenosis, the comprehensive cardiac workup, including coronary angiography, identified severe multi-vessel coronary artery disease as the likely underlying cause of his presentation and left ventricular dysfunction. This highlights the importance of a thorough and multidisciplinary approach in evaluating patients with complex cardiovascular presentations.

Although multi-vessel coronary artery disease was identified as the principal etiology in this case, differential diagnosis of flash pulmonary edema encompasses several cardiogenic mechanisms, including hypertensive crisis, valvular heart disease, and left ventricular diastolic dysfunction. These entities serve not only as potential diagnostic alternatives but also as established predisposing and precipitating factors for flash pulmonary edema. The diagnostic challenge is compounded by the high prevalence of cardio-renal risk factors in flash pulmonary edema patients, which frequently leads to diagnostic delays. Clinical observations indicate that patients typically experience 2-3 episodes of acute dyspnea before receiving a definitive flash pulmonary edema diagnosis [3]. Notably, the presence of preserved left ventricular systolic function and angiographically normal coronary arteries led to the consideration and evaluation for non-cardiac causes of dyspnea, including renal artery stenosis [8].

The initial suspicion of a renal artery stenosis was raised due to the patient's clinical presentation with sudden-onset flash pulmonary edema as well as malignant hypertension. A renal Doppler ultrasound is a non-invasive screening tool that can suggest the presence of renal artery stenosis by detecting increased blood flow velocities in the renal arteries [9,10]. However, it has limitations in terms of sensitivity and specificity, and further definitive imaging with angiography is often required when clinical suspicion remains high, with the advantage of intervention at the time of angiographic diagnosis [11].

In this case, even though the renal angiogram was planned pre-procedure due to the patient's presentation, following diagnostic cardiac catheterization, the indication for possible renal artery stenosis became strengthened with the finding of multi-vessel coronary artery disease, and a drive-by approach was also adopted during cardiac catheterization. Kelly reported that flash pulmonary edema is more common in patients with coronary artery disease than in patients with renovascular disease. In these patients, who are often elderly, there is a totally occluded vessel and collateral supply from another vessel with severe stenosis. Reduced flow to the collateral circulation or increased myocardial demand can then lead to a transient reversible myocardial ischemia, left ventricular dysfunction, and pulmonary edema [12]. This is a possible explanation for the pulmonary edema in this patient. It was interesting to find completely normal renal arteries in this patient, as previous reports suggest that patients with atherosclerotic disease in one bed are likely to have it in other beds [13].

This case underscores several important points. Firstly, it demonstrates the feasibility and successful execution of selective renal angiogram in Nigeria, expanding the availability of advanced diagnostic procedures for renovascular disease. Secondly, it highlights the necessity for definitive imaging when clinical suspicion warrants and renal ultrasonography is unavailable due to the limited availability of radiology specialists that sometimes exist in private hospitals of developing countries. Thirdly, it emphasizes the critical role of a comprehensive cardiovascular evaluation, including coronary angiography, in patients presenting with atypical symptoms like flash pulmonary edema, especially in those with multiple cardiovascular risk factors. Finally, it reinforces the importance of a multidisciplinary team approach involving nephrologists, cardiologists, and interventional specialists in optimizing the diagnosis and management of complex cardiovascular conditions.

## Conclusions

Flash pulmonary edema, often linked to bilateral renal artery stenosis and multi-vessel coronary artery disease, represents a critical diagnostic and therapeutic challenge. The successful management of this patient with coronary artery bypass grafting following the identification of severe coronary artery disease led to a positive outcome. This case contributes to the growing body of knowledge regarding the diagnosis and management of cardiovascular diseases in Nigeria. It also underscores the need for procedural versatility, particularly in the evaluation of alternative diagnoses, continued development, and accessibility of advanced diagnostic services.
